# The (Un)Clear Effects of Invalid Retro-Cues

**DOI:** 10.3389/fpsyg.2016.00244

**Published:** 2016-03-31

**Authors:** Marcel Gressmann, Markus Janczyk

**Affiliations:** ^1^Department of Psychology III, Julius Maximilians University of WürzburgWürzburg, Germany; ^2^Department of Psychology, Eberhard Karls University of TübingenTübingen, Germany

**Keywords:** visual working memory, retro-cue, attention, replication

## Abstract

Studies with the retro-cue paradigm have shown that validly cueing objects in visual working memory long after encoding can still benefit performance on subsequent change detection tasks. With regard to the effects of invalid cues, the literature is less clear. Some studies reported costs, others did not. We here revisit two recent studies that made interesting suggestions concerning invalid retro-cues: One study suggested that costs only occur for larger set sizes, and another study suggested that inclusion of invalid retro-cues diminishes the retro-cue benefit. New data from one experiment and a reanalysis of published data are provided to address these conclusions. The new data clearly show costs (and benefits) that were independent of set size, and the reanalysis suggests no influence of the inclusion of invalid retro-cues on the retro-cue benefit. Thus, previous interpretations may be taken with some caution at present.

## Introduction

Cues that validly direct attention to locations where relevant stimulation will appear soon thereafter improve performance. This has been known since the spatial pre-cuing experiments of Posner (1980), and cues also facilitate encoding of stimuli into visual working memory (VWM; e.g., Jiang et al., 2000; Janczyk and Reuss, 2016). Particularly interesting is that even retro-cues – cues that appear long after encoding has finished and when no additional sensory information is available – can improve performance if they validly indicate the item that is tested subsequently. Several studies have also included invalid retro-cues and assessed the performance costs induced by them (e.g., Astle et al., 2012b; Gözenman et al., 2014; Gunseli et al., 2015). The outcome, however, is less clear and some studies reported costs, others did not. Consequently, several authors arrived at the conclusion that there is some uncertainty about the existence of costs. In the present paper, we revisit two recent studies that employed invalid retro-cues and offered interesting conclusions based on their results. We believe, however, that other interpretations are possible and should be considered. As such, the present manuscript should rather be understood as a critical discussion of previous works’ findings instead of as a general critique of these works and approaches. Besides considering whether costs of invalid cues exist or not, we focus on the following two questions:

(1) Do costs depend on the number of learned items (i.e., on set size)? A recent study (Astle et al., 2012b) suggested that the existence of costs depends on set size and that accordingly different theoretical accounts apply for different set sizes. This interesting interpretation has been incorporated into framings and discussions of several recent studies (Pertzov et al., 2013; Backer and Alain, 2014; Li and Saiki, 2014; Matsukura et al., 2014; Gunseli et al., 2015). To address this question, we consider the original result sections of Astle et al. (2012b) and present data from a new experiment. This experiment included the most important conditions of Experiment 3 by Astle et al. (2012b) and may thus be seen as a conceptual replication. To foreshadow, results of this experiment do not support the original interpretation.
(2) Is the retro-cue effect (RCE) diminished or eliminated when invalid retro-cues are included? An affirmative answer was given by a recent study (Gözenman et al., 2014) for the case of a recall test. In our “Discussion” section, we provide a re-analysis of the original data in which we included a critical variable. Results of this analysis yield a negative answer though.

### The Benefit of Valid Retro-Cues

In a typical retro-cue task, participants are first presented with a set of to-be-learned items, for example, four colored circles arranged in a particular spatial way (learning screen). After a first delay (typically ≥1000 ms to exclude influences of iconic memory; Sperling, 1960) a cue – most often an arrow – is presented for a brief time and following a second delay of about 500 ms a (local) change detection task is applied where one colored circle and three annuli appear on screen (test screen).^1^ The participant’s task is to decide whether the same or a different color has been presented at the test location during the learning screen. The interesting manipulation concerns cue validity. While *valid cues* always indicate the exact location of the subsequently tested item, *neutral cues* are uninformative in this way, and change detection performance is better [more accurate and also often faster response times (RTs)] for valid cue trials compared to neutral cue trials (Griffin and Nobre, 2003; Landman et al., 2003). This performance difference is often called the RCE; in the present context – and because we will soon introduce the negative effect of invalid cues as “costs,” we use the term “benefit” for the performance difference between valid and neutral cues. The term RCE, in contrast, will be used broadly to refer to any impact of retro-cues. Retro-cue benefits appear stable across a variety of stimuli, testing procedures, timing parameters, and so on (e.g., Makovski and Jiang, 2007; Matsukura et al., 2007; Makovski et al., 2008; Astle et al., 2012b; Berryhill et al., 2012; Tanoue and Berryhill, 2012; Hollingworth and Maxcey-Richard, 2013; Janczyk and Berryhill, 2014).

### Costs of Invalid Retro-Cues?

Although several accounts have been proposed to explain RCEs, much of the literature still revolves around the prioritization and the protection account that were originally introduced and contrasted by Matsukura et al. (2007). Briefly, the *prioritization account* assumes that the cued item will be the first one that is compared to the item on the test screen and is thus given a head-start yielding the – on average – better performance with valid than with neutral cues (where no particular item is designated the first comparison). The *protection account*, in contrast, assumes that the cued item is protected from degradation via decay and/or interference from other VWM items or subsequent stimulation (see also Makovski et al., 2008) and therefore will remain more stable than other items which will degrade over time. (More details will be given in the “Discussion” section.) To distinguish these accounts, several recent studies employed *invalid retro-cues*, that is, cues that point to a position which will *not* be tested subsequently.

**Table 1** briefly summarizes studies with conditions that allow to assess benefits and costs separately:^2^

**Table 1:** Summary of studies that had included invalid retro-cues in their design.

Studies using change detection tasks	Exp.	*n*	Other variables	Benefit			Costs			Comments
				RT	PC	d′	RT	PC	d′	
Astle et al., 2012b	2	12	First delay	✓		✓	✓		✕	No benefit was observed for d′ with first delay
										of 9600 ms
	3	10	Set size 4	✓		✓	✓		✕	
			Set size 8	✓		✕	✓		✓	
	4	10	Set size 4	✓		✓	✓		✕	Pattern according to authors’ interpretation –
			Set size 8	✓		✕	✓		✓	See main text for elaborations
Astle et al., 2012a	1	12		✓		✓	✕		✓	Only adult group considered
Gözenman et al., 2014	1a	20	(w/o invalid)		✓			n/a		
	1b	20			✓			✕		
Griffin and Nobre, 2003	1	10		✓	✕		✕	✕		Only retro-cues conditions considered (according to Results sections)


	2	10		✓	✓		✕	✓		
Gressmann and Janczyk (this paper)		48	Set size 4	✓	✓		✓	✓		No interaction of cue type and set size


			Set size 8	✓	✓		✓	✓		
Li and Saiki, 2014	1	16				✓			✓	Only single-cueing conditions considered
	2	16				✓			✕	
	3	18				✓			✕	
Li and Saiki, 2015	1	16		✓		✓	✓		✓	Only retro-cues conditions considered
	2	24		✓		✓	✓		✓	
Rerko et al., 2014	2	24		✓	✓		✓	✓		
	3	22		✓	✓		✓	✓		
Shimi et al., 2014	2	19	(50% validity)	✕		✕	✕		✕	Only adult group considered

**Studies using recall tasks**	**Exp.**	***n***	**Other variables**	**Benefit**			**Costs**			**Comments**
				**err**	**pr**	**rp**	**err**	**pr**	**rp**	

Gözenman et al., 2014	2a	20	(w/o invalid)	✓			n/a			
	2b	20		✕			✕			No benefit according to authors’
										interpretation – See main text for elaborations
Gunseli et al., 2015		20	50% valid	✓	✓	✓	✓	✕	✕	Larger effects for the 80% valid condition
			80% valid	✓	✓	✓	✓	✓	✓	
Pertzov et al., 2013	1a	12		✓			✓			Benefit/costs increased across second delay
	1b	12		✓			✓			

*Benefits refer to the difference between valid and neutral/no-cue trials, costs indicate the difference between neutral/no-cue and invalid retro-cues. Note that the study by Matsukura et al. (2007) used invalid trials as well, but no neutral trials. Thus, it is not possible to assess costs and benefits separately. The gray shaded cells indicate the experiments that are discussed in detail in the main text. Benefits and costs for d′ in the study by Li and Saiki (2014) and for RTs and d’ in the study by Li and Saiki (2015) were significant according to personal communication with the authors. n = size of actually analyzed sample; PC, percent correct; RT, response time; err, raw angular error; pr, precision; rp, recall probability; w/o, without; n/a, not applicable*.

• *Benefits* (*valid* vs. *neutral cues*): When RTs were reported, all studies indicated a benefit in terms of RTs. Regarding accuracy or d′, the picture is a bit more mixed with two notable exceptions. Astle et al. (2012b) reported no benefit for set size 8 trials and Gözenman et al. (2014) reported no benefit when invalid trials were included in their Experiment 2b (we will come back to this finding in the “Discussion” section).
• *Costs* (*invalid* vs. *neutral cues*): Costs have been reported quite consistently for RTs with the exception of Experiments 1 and 2 in Griffin and Nobre (2003). In terms of accuracy or d′, reports are mixed and it was recently summarized that “observers tend to recognize invalidly cued items less accurately compared to neutrally cued items” (Matsukura et al., 2014, p. 1104). Gözenman et al. (2014, p. 1749) reached a similar conclusion and noted that there is a “lack of consistency with regard of the effects of invalid retro-cues on the RCE.” This mixed impression concerning costs may have to do with the fact that the effect of invalid retro-cues is weaker than that of valid ones. Thus, a lack of (significant) costs may be attributed to insufficient power of some studies. One particularly interesting pattern was again reported by Astle et al. (2012b), who reported costs only for set size 8 trials (but not for set size 4 trials). Thus, the pattern of benefits and costs appears to depend on set size (i.e., on VWM load). We suggest, however, that the data taken to support this interpretation are less clear, and we will provide elaborations on this in the next section.

### A Role of Set Size for Benefits and Costs?

In Experiment 1 of Astle et al. (2012b) set size 4 was used with valid and neutral cues and a benefit was reported for d’ and RTs for first delays from 150 to 9600 ms. In Experiment 2, invalid cues were included. For d′, there was a benefit for first delays of 150 and 1200 ms, but no costs (note that with a first delay of 9600 ms there was no RCE at all). In terms of RTs, both benefits and costs were reported. These results were taken as evidence for prioritization, but it was suggested that this might only apply if set size is within VWM capacity, because it would be detrimental to overall task performance if participants would use the cue to exclude items from VWM in this case (and, e.g., expose them to a higher decay rate). Yet, if set size exceeds VWM capacity, the cues may well be used to reduce VWM load by removing uncued items from VWM. To test this, set size was varied between two, four, and eight items in Experiment 3 (with eight items likely exceeding VWM capacity) and the first delay was chosen to ensure that the cues act on VWM and not on iconic memory (a random interval between 1500 and 2500 ms). In general, performance was worse with larger set sizes. The interesting pattern, however, relates to the interaction of cue type and set size on d′. For set size 4, that is, when VWM capacity was not exceeded, only benefits but no costs were observed. For set size 8, though – when capacity was exceeded – only costs were observed, suggesting that in fact retro-cues were used to free VWM from items. (Note that for set size 8 no benefit was observed.) Finally, Experiment 4 was run to replicate and extend this pattern by varying the first delay between 150 (tapping into iconic memory) and 1500 ms (tapping into VWM). The data of this experiment appear less clear though. First, in terms of d′, performance was worse for set size 8 compared with set size 4. Second, the main effect of cue type was decomposed into overall significant benefits and significant costs. Third, only the interaction between first delay and cue type (SOA and validity in the original paper) was significant. Subsequent decomposition of this interaction indicated benefits and costs for the short first delay of 150 ms. However, both costs and a small but significant benefit were also reported for the delay of 1500 ms. The most important point is: Because set size did not interact with other variables, this means that costs and benefits occurred at both set sizes. In other words, in contrast to Experiment 3 (1) a benefit was observed for set size 8 and (2) costs were observed for set size 4. Note that the absence of benefits with larger set sizes as reported from Experiment 3 is also at odd with a work of Makovski et al. (2008) where no difference in retro-cue benefits across set sizes of 2–6 items was reported.

Astle et al. (2012b) overall favored an explanation in terms of prioritization in combination with protection and load reduction if VWM capacity is exceeded. Such account is certainly interesting and was already incorporated into and mentioned by several recent publications (Pertzov et al., 2013; Backer and Alain, 2014; Li and Saiki, 2014; Matsukura et al., 2014; Gunseli et al., 2015). However, the account seems only be supported by the data of their Experiment 3, while the data from Experiment 4 is not in line with it. This situation, we believe, calls for new data to further investigate the validity of the original interpretation.

### The Present Experiment

In the following, we present an experiment that included the most important conditions of Astle et al.’s (2012b) Experiment 3 with a larger sample size (*n* = 48). Participants learned a display of four or eight colored circles and were tested with a local change detection task later.^3^ We used valid, neutral, and invalid cues and held the first delay constant. The second delay was systematically varied, though, to investigate degradation of items (see also Pertzov et al., 2013; Gözenman et al., 2014), and was extended to 2000 ms to enhance chances to find the interaction of cue type with set size. The main interest of this experiment was to test with more power whether or not the critical interaction of set size and cue type as observed in Astle et al.’s (2012b) Experiment 3 (but not Experiment 4) can be replicated. If yes and if we further follow their interpretation that with set size 8 the retro-cue is used to reduce VWM load, invalidly cued items should be subject to further degradation with increasing length of the second delay. This should not, however, be the case for set size 4. Considering the outcome of Astle et al.’s (2012b) Experiment 4, it is, however, not certain that we will find the critical interaction. Data analyses were guided by and focused on four critical hypotheses.

## Materials and Methods

### Participants

Forty-eight people from the Würzburg community participated (40 female; mean age 28.0 years; one left-handed) in two separated sessions of one hour each.^4^ They were naive as to the purpose of the experiment and reported normal or corrected-to-normal vision without deficits in color perception. All participants received either course credit or financial compensation (7cccc, per hour) for their participation. The study was conducted in accordance with the Declaration of Helsinki and the guidelines of the ethics committee at the University of Würzburg. All participants provided written informed consent prior to data collection.

### Stimuli and Apparatus

All stimuli were presented on a 19″ monitor. The stimuli were either four or eight circles (radius: 1.6 cm) of blue, brown, yellow, gray, green, purple, orange, or red color. Cues were either a white X (1.3 cm high; neutral cues) or white arrows (1.6 cm; valid and invalid cues). All stimuli were presented against a black background.

### Procedure and Design

The participants performed two similar experimental sessions. Each session started with an instruction and a brief practice block of ten trials, followed by five blocks of 96 trials each. In the middle and at the end of each block participants were given the opportunity of a short break. Cues were either neutral (thus uninformative), valid (the arrow pointed reliably to the position where the test-stimulus appeared later), or invalid (the arrow always pointed to another position). Each block consisted of 64 trials with valid cues (66.7%), 16 trials with invalid cues (16.7%), and 16 trials with neutral cues (16.7%). Thus, 80% of the informative cues were valid, and participants were instructed to use the cues to improve performance.

**FIGURE 1:**
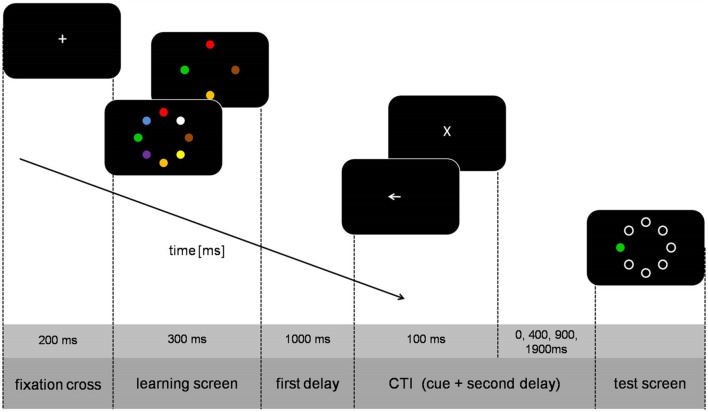
**Schematic illustration of trial sequence**.

The sequence of a trial is illustrated in **Figure 1**. Each trial started with the presentation of a fixation cross (0.8 cm high) for 200 ms after which the learning screen with four or eight circles appeared for 300 ms. The selection of the colors and their placement was chosen randomly on each trial. After a black screen (1000 ms) the cue was presented for 100 ms. The second delay from cue offset to test screen onset was either 0, 400, 900, or 1900 ms (translating to cue-test-intervals [CTI] of 100, 500, 1000, or 2000 ms). The test screen consisted of only one colored circle (test-stimulus) whereas the other circles were white annuli. In half of the trials color of the test-stimulus was identical to the color of the circle at the same position of the learning screen, whereas in the other half the colors were not identical. Participants gave their answer with the CTRL-keys of a standard QWERTZ keyboard (left key for ‘test-stimulus is identical,’ right key for ’test-stimulus is not identical’). After the response, the trial was finished and the screen turned black for 2000 ms.

### Analyses

As many other studies did, we analyzed accuracy (mean percent correct; PC) and mean RTs as dependent measures. Practice trials and the first block of Session 1 as well as trials with RT ≥ 10000 ms (0.03 %) were excluded for all analyses. For RT analyses, all erroneous trials and those where RTs deviated by more than 2.5 standard deviations from the respective cell means were excluded (separately for each participant and analyzed design cell). Three variables of interest were varied within participants: (1) set size: 4 vs. 8, (2) cue type: valid vs. neutral vs. invalid, and (3) second delay: 0 vs. 400 vs. 900 vs. 1900 ms. We initially grouped participants according to whether they performed with the slightly unequal trial frequencies or not (see also Footnote 4) and ran a mixed ANOVA with all three repeated measures and this grouping variable as an additional between-subjects variable. This grouping variable neither produced a main effect, PC: *F*(1,46) = 2.83, *p* = 0.099, $\eta_{p}^{2}$ = 0.06, RT: *F*(1,46) = 0.22, *p* = 0.642, $\eta_{p}^{2}$ = 0.01, nor did it interact with the other variables [all *F*s ≤ 2.61, all *p*s ≥ 0.054; more information and detailed test statistics are provided in Appendix 1 in the online supplemental data (Table A1)]. All subsequently reported analyses were therefore run on the total sample of 48 participants. Guided by the objectives of the present research we approached the data with different analyses and structured the Results section accordingly. Within each subsection, we first focus on PC results, which are then followed by RT results. This approach followed many other studies with the retro-cue paradigm and allows to exclude speed-accuracy trade-offs.

## Results

### Analysis 1

Data were first submitted to an ANOVA including all three repeated measures. We expected worse performance for set size 8 than for set size 4 and an influence of cue type on performance that might not be visible at the shortest second delay of 0 ms (Tanoue and Berryhill, 2012; Pertzov et al., 2013), thus an interaction of cue type and second delay. Against the background of the account of Astle et al. (2012b) and assuming increasing degradation for items that were removed from VWM, a significant three-way interaction can be expected. This interaction should be driven by different consequences of invalid cues in both set size conditions with an increasing second delay.

**FIGURE 2:**
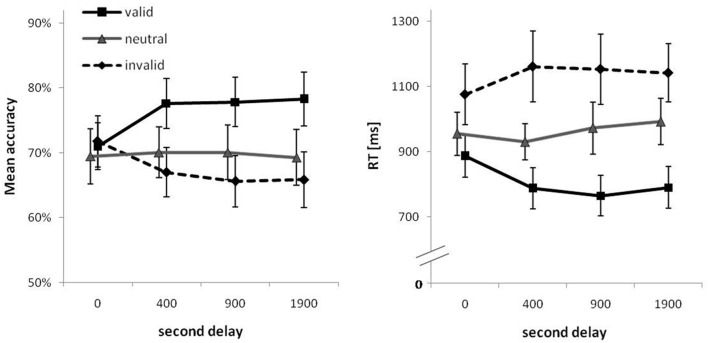
**Mean percent correct (PC; Left) and mean correct response times (RTs; Right) as a function of second delay and cue type.** Error bars are 95% confidence intervals for each data point.

Descriptive statistics for both PC and RTs are summarized in **Table 2** and are visualized in **Figure 2**. Detailed test statistics from the ANOVA are summarized in **Table 3**. In terms of PC, performance was better for set size 4 trials (82%) compared to set size 8 trials (65%) and in trials with valid cues (76%) compared to trials with neutral cues (70%) and invalid cues (68%). Thus, an RCE was present in this experiment. The RCE was not modulated by set size, but the interaction of cue type and second delay was significant (see **Figure 2**, left). The interaction of set size and second delay approached significance, but the three-way interaction was not significant. Descriptively it seems as if the RCE does not appear until a second delay of 400 ms after which it remains stable. In general, a very similar picture emerged for RTs. RTs were longer for set size 8 (908 ms) than for set size 4 trials (862 ms). Further, fastest responses were given to valid cues (805 ms), RTs were intermediate to neutral cues (967 ms), and slowest to invalid cues (1133 ms). Like for accuracy, the interaction between cue type and second delay was significant indicating a growing cue impact up to a second delay of 400 ms (see **Figure 2**, right). The three-way interaction was not significant.

**Table 2:** Descriptive statistics: mean percent correct and mean correct response times (in ms) as a function of set size, cue type, and second delay duration.

		Percent correct				Response time [ms]			
		Second delay [ms]				Second delay [ms]			
Set size	Cue type	0	400	900	1900	0	400	900	1900
4	Valid	79.1	86.3	87.2	87.2	869	758	744	771
	Neutral	76.2	78.3	78.1	78.7	925	928	959	987
	Invalid	78.5	74.4	75.0	74.3	1053	1136	1094	1105
8	Valid	63.0	69.2	68.3	69.4	908	827	787	812
	Neutral	63.1	62.5	62.3	60.6	996	945	1002	1012
	Invalid	65.0	58.2	57.3	57.5	1100	1194	1222	1195

**Table 3:** Detailed test statistics for the three-way ANOVA on mean percent correct and response times as dependent measures (Analysis 1 in the main text).

	Percent correct				Response times			
Effect	*F* (df_n_,df_m_)	*p*	$\eta_{p}^{2}$	𝜀	*F*(df_n_,df_m_)	*p*	$\eta_{p}^{2}$	𝜀
Set size	199.01 (1,47)	<0.001	0.81		16.55 (1,47)	<0.001	0.26	
Cue type	61.77 (2,94)	<0.001	0.57	0.88	146.84 (2,94)	<0.001	0.76	0.72
Second delay	0.35 (3,141)	0.787	0.01		0.58 (3,141)	0.596	0.01	0.82
Cue type × set size	0.89 (2,94)	0.413	0.02		1.97 (2,94)	0.155	0.04	0.79
Cue type × second delay	11.69 (6,282)	<0.001	0.20	0.78	15.53 (6,282)	<0.001	0.25	0.72
Set size × second delay	2.57 (3,141)	0.056	0.05		0.50 (3,141)	0.656	0.01	0.85
Set size × cue type × second delay	0.28 (6,282)	0.945	0.01		1.43 (6,282)	0.226	0.03	0.68

*df_n_ and df_m_ denote the numerators’ and denominators’ degrees of freedom for the respective F-test. 𝜀 is the Greenhouse–Geisser estimation of sphericity violations*.

### Analysis 2

To follow-up and strengthen Analysis 1, the data from the invalid cue trials were submitted to a 2 (set size) × 4 (second delay) ANOVA with Helmert contrasts on the second delay variable. Interactions are again expected if the invalid cue only in set size 8 trials led to an exclusion of items from VWM and their subsequent degradation. As part of this analysis, we also compared the accuracy on invalid set size 8 trials against chance level to ensure that an absent effect of second delay was not due to floor effects.

Accuracy was higher for set size 4 than for set size 8, *F*(1,47) = 112.72, *p* < 0.001, $\eta_{p}^{2}$ = 0.71, and showed a drop from the second delay of 0 ms to later levels, *F*(1,47) = 21.31, *p* < 0.001, $\eta_{p}^{2}$ = 0.31, for the first Helmert contrast. Performance level then remained rather stable and none of the later contrasts was significant, all *F*s < 1, *p*s ≥ 0.837, all $\eta_{p}^{2}$ ≤ 0.01. Importantly, the pattern was the same for both set size conditions and no interactions of Helmert contrast × set size were significant, all *F*s ≤ 2.80, all *p*s ≥ 0.101, all $\eta_{p}^{2}$ ≤ 0.06. A similar picture was evident for RTs. RTs were longer with set size 8 than with set size 4, *F*(1,47) = 9.99, *p* = 0.003, $\eta_{p}^{2}$ = 0.18. The first Helmert contrast (second delay 0 ms vs. later) was significant, indicating an effect of the (invalid) retro-cue between the 0 and the 400 ms second delay, *F*(1,47) = 10.95, *p* = 0.002, $\eta_{p}^{2}$ = 0.19. None of the later contrasts was significant though, suggesting that performance was relatively constant from the 400 ms second delay on, all *F*s < 1, all *p*s ≥ 0.613, all $\eta_{p}^{2}$ ≤ 0.01. Importantly, the pattern of performance was similar for both set size conditions and no interactions of Helmert contrast × set size were significant, all *F*s ≤ 1.48, all *p*s ≥ 0.230, all $\eta_{p}^{2}$ ≤ 0.03.

Finally, the mean accuracies for invalid cues at set size 8 were all higher than chance (50%), all *p*s ≤ 0.001. Hence, a further decrease in performance for invalid trials across the varying second delays was possible in principle.

### Analysis 3

The data from each second delay level were submitted to 2 (set size) × 3 (cue type) ANOVAs with repeated contrasts on the variable cue type. The first comparison indicates costs (invalid vs. neutral) and the second comparison indicates benefits (neutral vs. valid). The interesting question of this analysis relates again to interactions, which can be expected as significant against the background of Astle et al.’s (2012b) Experiment 3. In particular, a pattern should then emerge with only a benefit for set size 4 and only costs for set size 8.

**FIGURE 3:**
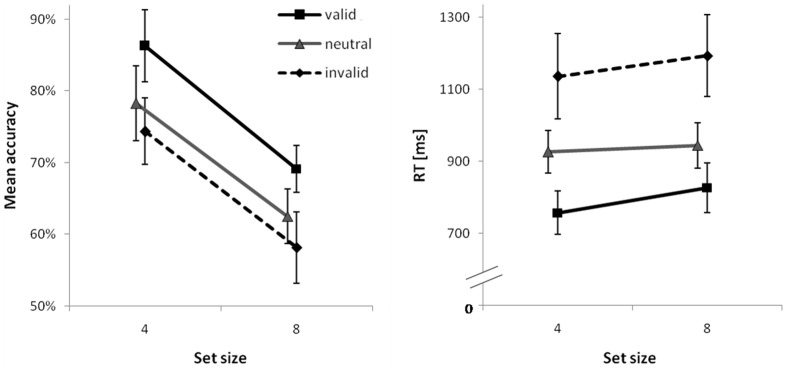
**Mean percent correct (PC; Left) and mean correct response times (RTs; Right) for the second delay of 400 ms as a function of set size and cue type.** Error bars are 95% confidence intervals for each data point.

Detailed test statistics for this analysis are summarized in **Table 4** and descriptive statistics are visualized for the 400 ms second delay in **Figure 3**. Accuracy was higher for set size 4 than for set size 8. For the 0 ms second delay neither significant costs nor a benefit was observed; however, both were consistently evident at the higher second delay levels, even though effect sizes for costs were smaller than those for benefits. Both costs and benefits were comparable for both set size conditions and no interactions of contrast × set size were significant. A very similar picture emerged for RTs. The only difference was the one and only significant interaction of set size and the benefit-contrast at the 400 ms second delay level suggesting a slightly smaller RT benefit for set size 8 than for set size 4.

**Table 4:** Detailed test statistics from the ANOVAs on mean percent correct and response times at each second delay level separately (Analysis 3 in the main text).

			Percent correct			Response times		
Second delay	Effect	Contrast	*F*(1,47)	*p*	$\eta_{p}^{2}$	*F*(1,47)	*p*	$\eta_{p}^{2}$
0 ms	Set size		104.33	<0.001	0.69	14.49	<0.001	0.24
	Cue type	Invalid vs. neutral (costs)	3.08	0.086	0.06	18.17	<0.001	0.28
		Neutral vs. valid (benefit)	1.44	0.237	0.03	30.53	<0.001	0.39
	Set size × cue type	Invalid vs. neutral (costs)	0.03	0.873	<0.01	0.55	0.464	0.01
		Neutral vs. valid (benefit)	1.75	0.192	0.04	2.07	0.157	0.04
400 ms	Set size		112.75	<0.001	0.71	7.43	0.009	0.14
	Cue type	Invalid vs. neutral (costs)	6.93	0.011	0.13	51.12	<0.001	0.52
		Neutral vs. valid (benefit)	32.94	<0.001	0.41	76.35	<0.001	0.62
	Set size × cue type	Invalid vs. neutral (costs)	0.02	0.882	<0.01	0.85	0.360	0.02
		Neutral vs. valid (benefit)	0.41	0.526	0.01	4.18	0.046	0.08
900 ms	Set size		166.04	<0.001	0.78	15.02	<0.001	0.24
	Cue type	Invalid vs. neutral (costs)	6.49	0.014	0.12	63.30	<0.001	0.57
		Neutral vs. valid (benefit)	37.99	<0.001	0.45	123.42	<0.001	0.72
	Set size × cue type	Invalid vs. neutral (costs)	0.43	0.517	<0.01	3.17	0.082	0.06
		Neutral vs. valid (benefit)	1.46	0.234	0.03	<0.01	0.995	<0.01
1900 ms	Set size		150.03	<0.001	0.76	4.69	0.035	0.09
	Cue type	Invalid vs. neutral (costs)	5.76	0.020	0.11	35.82	<0.001	0.43
		Neutral vs. valid (benefit)	43.82	<0.001	0.48	121.90	<0.001	0.72
	Set size × cue type	Invalid vs. neutral (costs)	0.25	0.617	<0.01	1.66	0.204	0.03
		Neutral vs. valid (benefit)	0.02	0.884	<0.01	0.19	0.668	<0.01

*Repeated contrasts were calculated on the variable cue type to assess benefits and costs*.

### Analysis 4

Finally, the prioritization account predicts similar RTs in trials with valid cues for both set size conditions, but in trials with neutral cues slower responses for set size 8. To test this, we compared the RTs between set size 4 and 8 for valid and neutral cues at each of the second delay levels where an RCE can be expected (second delay ≥ 400 ms).

For valid cues, all paired *t*-test were significant (all *p*s ≤ 0.013) with slower responses for set size 8 than for set size 4 trials. For neutral cues, no significant differences were found (all *p*s ≥ 0.142).^5^ This is (almost) the opposite outcome of what a pure prioritization account would predict.

## Discussion

Participants in our experiment worked on a local change-detection task with either set size 4 or 8. During the retention interval, a valid, neutral, or invalid retro-cue was presented and the second delay from cue to test screen was systematically varied. Although the results replicate several findings they fail to reveal an interaction of set size and cue type.

### Summary of Results

In line with previous studies, the RCE needed some time to fully develop (Pertzov et al., 2013). In terms of PC, no impact of the retro-cue was evident at the shortest second delay of 0 ms but only at the longer second delays. From then on, performance remained relatively constant (Tanoue and Berryhill, 2012). RTs behaved in general similar, although a small RCE was observable even at the 0 ms second delay. Further, once the retro-cue affected performance, we consistently observed both benefits and costs, although the latter were smaller in terms of effect sizes. Thus, a first finding is that costs exist, but may have been unnoticed due to insufficient power in some previous studies (see **Table 1**).

The second important finding is that benefits and costs were independent of the set size manipulation. In fact, there was no consistent sign of an interaction between set size and cue type as reported in Experiment 3 of Astle et al. (2012b).^6^ This finding, however, conforms to the reports of Makovski et al. (2008) who did also not find an interaction of set size and the RCE. To further assess the strength of evidence favoring the null hypothesis of the critical interaction, we calculated the Bayes factor and the probability of the null hypothesis (Wagenmakers, 2007; Masson, 2011). The resulting values are summarized in **Table 5**. Following Raftery (1995), this evidence can be interpreted as strong, except for one case (RT as dependent measure, second delay of 900 ms) where it is positive evidence. Finally, the stable performance with second delays of ≥ 400 ms was also observed for trials with invalid cues, and is not in line with degradation of items via, for example, temporal decay. Importantly, this was not even observed in set size 8.^7^

**Table 5:** Bayes factors (BF) and posterior probabilities for the null hypothesis (*p*(H0|data)) according to Masson (2011) for the interaction of set size and cue type (calculations based on *n* = 48).

	Percent correct		Response times	
Second delay	BF	*p*(H0|data)	BF	*p*(H0|data)
0 ms	29.98	0.969	33.44	0.971
400 ms	45.06	0.978	28.00	0.966
900 ms	33.82	0.971	10.93	0.916
1900 ms	44.78	0.978	26.00	0.963

In sum, these results indicate that (1) benefits *and* costs are reliable phenomena and (2) are *not affected by set size* variations; at least not in a setup like ours with valid, neutral, and invalid retro-cues. Thus, the mechanisms giving rise to RCEs likely do not depend on the amount of memorized material. This was the question raised in Point 1 of the “Introduction” Section. We will now turn to Point 2 and address another recent finding made with the inclusion of invalid retro-cues (Gözenman et al., 2014).

### Is the Benefit Reduced or Eliminated When Invalid Cues are Included?

Gözenman et al. (2014) argued that the protection account predicts diminished benefits when invalid retro-cues are included compared to conditions without invalid retro-cues. In Experiment 1, a local change detection task with set size 4 was used and the second delay varied from 100 to 24000 ms. In Experiment 1a (*n* = 20), only valid and neutral cues were employed; in Experiment 1b (*n* = 20) additional invalid cues were used. Analyzed individually, Experiment 1a revealed a benefit in terms of PC and decreasing PC with longer second delays. The significant interaction was due to the fact that the effect of second delay was only found for neutral trials (thus, the benefit increased with longer delays). In Experiment 1b, only a benefit (“*p* = 0.05”, p. 1750) but no costs were observed (“*p* = 0.41”, p. 1750). The comparison of the benefits in Experiments 1a and 1b yielded an effect of second delay, but – most importantly – neither a main effect of experiment nor a significant interaction. In other words, the benefit was of similar size in both experiments. Experiments 2a and 2b followed the same reasoning but (1) a recall task where participants had to recall and reproduce the spatial orientation of a line at the tested location (see also Pertzov et al., 2013) and (b) second delays only from 800 to 10000 ms were used. In Experiment 2a, valid cues yielded more precise recall than neutral cues did (i.e., a benefit), and recall precision was reduced with longer second delays. In Experiment 2b, precision also became less with longer second delays. There was also a significant main effect of cue type, but notably, no benefits (“*p* = 0.305”, p. 1752) but marginally significant costs (“*p* = 0.065”, p. 1752) were reported. The interaction of second delay and cue type was not significant. Based on these results the authors’ concluded that in Experiment 2 “the presence of invalid retro-cues significantly reduced the RCE” (p. 1752).

Critically, differences in the size of the benefit were actually not tested because no between-experiment comparison was reported for Experiment 2. However, this is necessary to make any conclusions about differences in the size of the benefits in Experiments 2a and 2b. The non-significant benefit^8^ in Experiment 2b itself does not speak to its descriptive size in comparison to Experiment 2a (see, e.g., Cantor, 1956, or Nieuwenhuis et al., 2011, for elaborations on this issue). Reanalyses of the data in fact revealed a significant main effect of cue type, thus a benefit, but no interaction with the experiment-variable.^9^ Thus, even though this latter result hinges on retaining a null hypothesis, there is no indication that the RCE was smaller in Experiment 2b than in 2a – essentially the same result as in Experiment 1 of Gözenman et al. (2014). As such, this experiment did in fact replicate the benefit observed by Pertzov et al. (2013). What was not replicated though was the interaction with the second delay manipulation. The reasons for this are unclear, but the very different scales across which second delay was manipulated in both studies prevent a direct comparison.

It is important, however, to note that we do not consider the entire issue completely resolved. In particular, a recent study highlighted that the ratio of valid to invalid trials may be important (Gunseli et al., 2015) and affects the size of the RCE. Results from this study indicate that the RCE is overall larger and costs only occur with high retro-cue reliability (e.g., a ratio of 4:1) compared to low reliability (e.g., a ratio of 1:1). The ratio in our experiment was 4:1 and thus our results fit with this observation. Note also that the ratio in the study of Gözenman et al. (2014) was 2:1 and no costs were reported. Costs (but also benefits) were absent as well in Experiment 2 of Shimi et al. (2014) with a ratio of 1:1. Against this background, a larger benefit would make sense in the Gözenman et al. (2014) study without invalid retro-cues (where cue validity was 100%) compared to the conditions with invalid retro-cues (where cue validity was only 66%). Frankly, we cannot offer a definite solution at present but only offer speculations. One reason for this discrepancy might be that participants in the Gunseli et al. (2015) study were always confronted with invalid retro-cues and performed in both validity conditions, whereas in the Gözenman et al. (2014) study, the inclusion of invalid retro-cues (and thereby the manipulation of cue validity) was implemented between-participants.

### Explanations for RCEs

As noted in the Section “Introduction,” much of the retro-cue literature and research revolves around the prioritization and the protection account. What do the present findings and analyses mean for them?

According to the prioritization account, which was first suggested by Matsukura et al. (2007), the retro-cue is used to prioritize one item to begin with in the memory test thereby eliminating the requirement for an exhaustive memory search. (Please note that Matsukura et al., 2007 did not observe evidence for this account and preferred the protection account instead.) Because “all memoranda are subject to the same decay profiles” (Gözenman et al., 2014, p. 1749), all items will be equally available in VWM (at least as long as capacity is not exceeded). Assuming that there is no time-related degradation of VWM representations (e.g., via temporal decay), “a pure prioritisation account, in which cued and uncued items are equally available at probe onset, would predict cue validity effects on RTs but not on accuracy” (Astle et al., 2012b, p. 147). Two arguments can be made against a crucial role of prioritization in experiments like ours. First, with a local change detection task (arguably the most often employed test) the one comparison that needs to be made is entirely determined by the single (colored) object on the test screen and thus no additional prioritization is necessary (see also Souza et al., 2014). In fact, Makovski et al. (2008, p. 374, Experiment 2) used a local change detection task and argued that because “only a single comparison needs to be made, the simplified-comparison account [which comes close to the prioritization account; added by MG and MJ] predicted that the retro-cue should no longer enhance performance.” Second, following the reasoning above, no costs in terms of accuracy should occur (at least not for set size 4), but clearly they did in our experiment and in other studies (cf. **Table 1**; e.g., Astle et al., 2012a; Li and Saiki, 2015). To account for accuracy costs, one needs to incorporate (rapid) temporal decay by which items become degraded when re-prioritization becomes necessary in case of invalid cues. While this issue is heavily debated, evidence against temporal decay exists (e.g., Lewandowsky et al., 2009; Shipstead and Engle, 2013). If there was prioritization in our task, however, the results of Analysis 4 do not comply with the predictions and provide further evidence against prioritization. That said we can nonetheless not certainly exclude a role of prioritization, for example, in global change detection tasks.

The protection account, in contrast, assumes that a cued item is protected from further degradation via temporal decay (Matsukura et al., 2007) and/or interference from other VWM items, subsequent stimulation at the time of test (Makovski et al., 2008), or during the retention interval (e.g., Pinto et al., 2013). As a consequence, its representation remains more stable than that of other items. The original evidence provided by Matsukura et al. (2007) was criticized later for relying on indirect evidence of only small magnitude (e.g., Pertzov et al., 2013; Gözenman et al., 2014). Pertzov et al. (2013) aimed to provide direct evidence for the protection hypothesis. They used a recall task and did not observe diminishing accuracy with increasing second delays (0–3000 ms) for valid trials, but only for neutral and even more so for invalid trials. In contrast, Gözenman et al. (2014) concluded that no benefit was evident when including invalid retro-cues in their Experiment 2b (also with a recall task). Our reanalysis in the preceding section, however, arrives at the opposite conclusion and suggests that a benefit (and thus an impact of retro-cues) was replicated in their experiment and the benefit was of the same size whether or not invalid retro-cues were included. Following their argument, this counts as evidence against protection. Moreover, an interaction of cue type and second delay (800–10000 ms) was not found and the performance drop was similar for valid, neutral, and invalid trials from the second delay of 3000 to 10000 ms. This again casts some doubt on the protection account, even though the different spans of second delay durations in this study and the one by Pertzov et al. (2013) prevent a direct comparison^10^.

In sum, there seems to be not much of consistent evidence for a crucial role of attention in protecting one particular item from further degradation. Further doubts come from recent studies showing that sustained (focal) attention on the cued item is not necessary to produce a retro-cue benefit (Hollingworth and Maxcey-Richard, 2013; Rerko et al., 2014). Thus, even when attention is drawn away from the cued item in VWM, it remains in a highly accessible state to which subsequent retrieval can resort (Rerko and Oberauer, 2013; see also Großer and Janczyk, 2014; further, Janczyk et al., 2008, observed a similar finding for verbal working memory with updating tasks [see Garavan, 1998, or Janczyk and Grabowski, 2011]).

One recent study suggested a somewhat different view on the effect of retro-cues that could perhaps accommodate our findings (Souza et al., 2014). According to this account, retro-cues are used to free working memory from (temporarily) irrelevant items, more or less irrespective of how much material has been encoded initially (“removal hypothesis”). Once outsourced from central working memory to activated long-term memory (e.g., Oberauer, 2002), these items do not interfere with selection of one particular item by the focus of attention at the memory test. However, if tested subsequently, retrieval from there is less precise and slower than from inside central working memory, thereby producing the observed costs (see also Rerko et al., 2014). Such an account is also supported by other findings. For example, Williams and Woodman (2012, Experiment 3) used the CDA (contralateral delay activity) component of the ERP (event related potential) in an experiment where a retro-cue either indicated to-be-remembered or the to-be-forgotten memory items. In both conditions a CDA was evident, however, with its onset and amplitude being larger for the remember condition than for the forget condition. This latter observation led the authors to suggest that it might indicate the removal of irrelevant items from VWM. In a follow-up study this possibility was further investigated, and it was concluded that a forgetting cue makes people discard the cued items from VWM (Williams et al., 2013). Note, however, that a significant interaction of set size (2 vs. 4 vs. 6) and cue type (valid vs. no-cue) provided important evidence for the removal hypothesis of Souza et al. (see their Experiment 1). This contrasts with our findings (and that of Makovski et al., 2008), and causes some doubts about the role of removal in our present experiment. One possible reason for this discrepancy might be the reduced validity of the retro-cues. Instead, in this case, the retro-cues may have been used to strengthen the cued items representation (see also Souza et al., 2015).

To further shed some light on this, we analyzed Cowan’s K (Cowan, 2001) collapsed for the second delays of 400 and 900 ms as a function of set size and cue type, with repeated contrasts on the factor cue type. If a retro-cue is used to remove items from VWM, one would expect smaller *K*-values for invalid than for neutral cues, because following a neutral trial nothing special happens but participants try to remember as much items as possible, while with an invalid cue VWM is purposefully freed from items other than the cued one. *K*-values were 3.2, 3.4, and 3.8 for set size 4 and 4.8, 5.3, and 6.7 for set size 8 trials (invalid, neutral, and valid cues, respectively). Thus, *K*-values were larger for set size 8 trials, *F*(1,47) = 91.69, *p* < 0.001, $\eta_{p}^{2}$ = 0.66, and larger for valid than for neutral than for invalid retro-cues, *F*(2,94) = 27.93, *p* < 0.001, $\eta_{p}^{2}$ = 0.37, for the overall effect of cue type. The interaction was also significant, *F*(2,94) = 12.95, *p* < 0.001, $\eta_{p}^{2}$ = 0.22, pointing to a larger retro-cue benefit for set size 8 trials than for set size 4 trials. On the one hand, this finding seems more in line with the results of Souza et al. (2014) than with those of Astle et al. (2012b). On the other hand, we are hesitant to interpret this finding unambiguously, because the corresponding *K*-values can also only show that the cued item was not removed and if accuracy is high then, and multiplied by set size, this gives the larger *K*-values for valid cues in set size 8. Critically, *K*-values seem to be smaller for invalid compared to neutral cues, *F*(1,47) = 4.18, *p* = 0.047, $\eta_{p}^{2}$ = 0.08, and this contrast did not interact with set size, *F*(1,47) = 0.34, *p* = 0.560, $\eta_{p}^{2}$ < 0.01.^11^ Given these latter analyses, removal did happen in both set size conditions, but likely did not play a large role (perhaps only in few trials) and as a consequence left only minor traces in the data.

Although the present study was not designed to distinguish the various accounts for the RCE, it still helps to constrain them. In light of the present data and the considerations above, the account of Souza et al. (2014) appears most promising to us. We concede, however, that most likely several accounts are not mutually exclusive and their application may depend on one or more experimental variables. Because the various accounts are typically thought to be distinguished by their predictions concerning the costs of invalid cues, also their impact may depend on one or more experimental variables. Based on our experiment, we suggest that set size (or VWM load) is not critical.

## Conclusion

The purpose of this research was to revisit two recent findings on the effect of invalid retro-cues^12^. Our experiment and literature analysis showed that the typical benefit of valid retro-cues, but also costs of invalid retro-cues, are observed in terms of RTs and accuracy. Importantly, this pattern does not depend on set size, and thus previous interpretations of published data may be taken with caution.

## Author Contributions

MG and MJ planned and designed the research, MG performed research, MG and MJ analyzed data and wrote the paper.

## Conflict of Interest Statement

The authors declare that the research was conducted in the absence of any commercial or financial relationships that could be construed as a potential conflict of interest.

## Footnotes

1 We distinguish local change detection (where only one stimulus occurs at test, either centrally or peripherally) from global change detection where a whole stimulus array is presented at test again, and a change can potentially occur at each single location.

2 In this context, we will not discuss Experiment 2 of Shimi et al. (2014) in which neither benefits nor costs were observed. This, however, was due to the ratio of valid:invalid cues being 1:1. Thus, cues were effectively uninformative.

3 The original Experiment 3 of Astle et al. (2012b) also employed a set size 2 condition, but the interesting pattern was evident in differences between set size 4 and 8 only. Further, in their Experiment 4 only these set size conditions were realized and we thus decided to omit set size 2 from the very beginning.

4 Data of the first 24 participants were collected with an experimental program with a slight typing mistake. The different combinations of the three variables cue type, set size, and second delay were intended to be repeated equally often, but due to the typing mistake they occurred with slightly unequal frequencies. The intended number of trials was 18 for invalid and neutral cues and 72 for valid cues (per combination of set size and second delay). For participants 1–24, the minimum and maximum median of trials were 13 and 23.5 for invalid and neutral cues (ranging from 9 to 31 for individual participants), and 69 and 75 for valid cues (ranging from 61 to 82 for individual participants). We decided to continue testing until data of the first half of the intended sample size was collected. As explained later in detail, grouping the participants according to whether they belong to the participants with the slightly unequal frequency of trial combinations or not did not interact with any other variables.

5 When additionally considering the second delay = 0 ms data, there was one exception with faster responses for set size 4 trials (*p* = 0.002).

6 To be precise, there was one significant interaction for RTs. Because this was the only finding in this direction we consider it a chance finding instead of interpreting an effect that was not once replicated despite the multiple possible opportunities in these analyses (see **Table 4**).

7 We ran another experiment (*n* = 48) with only set size 8 trials where the second delay was extended to 4900 ms. In this experiment, no performance difference was observed for invalid cue trials from 1900 to 4900 ms second delay.

8 This finding might also have to with the fact that in Experiment 2b less trials with valid and neutral cues were used (78 trials) compared to Experiment 2a (105 trials each). One consequence of this might be less precise point estimations in Experiment 2b than in Experiment 2a thereby blurring the effect of cue type in Experiment 2b.

9 We thank Filiz Gözenman for providing us the data from their study. We submitted the data from their Experiment 2 to an ANOVA with cue type (valid vs. neutral) and second delay (800 vs. 3000 vs. 10000 ms) as repeated measures and experiment (2a vs. 2b) as a between-participants variable. Experiment exerted no main effect, *F*(1,38) = 1.98, *p* = 0.168, $\eta_{p}^{2}$ = 0.05, but second delay, *F*(2,76) = 38.16, *p* < 0.001, $\eta_{p}^{2}$ = 0.50, and cue type did, *F*(1,38) = 10.25, *p* = 0.003, $\eta_{p}^{2}$ = 0.21. The latter result reflects a benefit. The most important result for the present purposes relates to the interaction of experiment and cue type, which was far from being significant, *F*(1,38) = 0.17, *p* = 0.686, $\eta_{p}^{2}$ < 0.01. In other words, the benefit was significant and of the same size in both Experiments 2a and 2b. The three-way interaction was not significant, *F*(2,76) = 1.54, *p* = 0.222, $\eta_{p}^{2}$ = 0.04. Of less importance to the present purposes, there was further a significant interaction of experiment and second delay, *F*(2,76) = 4.82, *p* = 0.011, $\eta_{p}^{2}$ = 0.11, and the interaction of cue type and second delay was not significant, *F*(2,76) = 2.40, *p* = 0.098, $\eta_{p}^{2}$ = 0.06.

10 In fact, in Experiment 1a in Gözenman et al. (2014) such an interaction was found for cue type (valid vs. neutral) and second delay when using a local change detection task. In Experiment 1b, however, the interaction was absent and valid, neutral, and invalid trials produced diminished performance (in terms of accuracy) from the second delay of 2400 to 24000 ms.

11 The rationale for using the second delays of 400 and 900 ms only was that this range closely matches the one used by Astle et al. (2012b). Including the second delay of 1900 ms additionally did not alter the overall picture, but we acknowledge that the contrast comparing neutral and invalid retro-cues just failed to exceed the conventional significance criterion, *F*(1,47) = 3.79, *p* = 0.057, $\eta_{p}^{2}$ = 0.08; interaction with set size: *F*(1,47) = 0.24, *p* = 0.630, $\eta_{p}^{2}$ < 0.01.

12 Please note that we do not claim that our experiment is an exact replication of the original experiment. There are, of course, differences in the design and procedure that are sometimes unavoidable (other rooms, other experimenters, …) and sometimes intended (e.g., slightly other cue visualizations, a different first delay,…). We believe though that none of these factors should alter an outcome that leads to universally valid interpretations. We admit that this might not be everybody’s opinion and one may attribute the different outcomes to these differences in experimental details. In this case, however, the original interpretation is restricted to one particular design and stimulus arrangement.
