# Supplementary material for: The (Un)Clear Effects of Invalid Retro-Cues
Source: Front Psychol. 2016 Mar 31;7:244. doi: 10.3389/fpsyg.2016.00244 (PMC4815295; doi:10.3389/fpsyg.2016.00244)
Supplement: Supplementary file 1 [file Table_1.DOCX]

**Appendix A**

Table A1 summarizes test statistics of Analysis 1 when additionally including the grouping variable (participants 1-24 vs. 25-48; referred to as ‘group’). The almost significant test involving the factor group mentioned in the main text concerns the interaction set size × second delay × group. To follow-up on this, three-way ANOVAs were calculated for both groups separately. The interaction set size × second delay was significant for participants 1-24 (Group 1), *F*(3,69) = 3.37, *p* = .023, η_p_² = .13, but not for participants 25-48, *F*(3,69) = 1.64, *p* = .189, η_p_² = .07. This difference does, however, not undermine our main points as they are referring mostly to interactions of setsize and cue type.

**Table A1** Detailed test statistics for the mixed ANOVA with all three repeated measures and the grouping variable experiment as an additional between-subjects factor to test the consequence of the typing error in the first half of the sample.

|  | percent correct | | | | response times | | | |
| --- | --- | --- | --- | --- | --- | --- | --- | --- |
| effect | *F* (df_n_,df_m_) | *p* | η_p_^2^ | ε | *F*(df_n_,df_m_) | *p* | η_p_^2^ | ε |
| group | 2.83 (1,46) | .099 | .06 |  | 0.22 (1,46) | .642 | .01 |  |
| set size | 197.09 (1,46) | < .001 | .81 |  | 16.52 (1,46) | < .001 | .26 |  |
| cue type | 62.40 (2,92) | < .001 | .58 | .87 | 144.81 (2,92) | < .001 | .76 | .72 |
| second delay | 0.35 (3,138) | .786 | .01 |  | 0.58 (3,138) | .597 | .01 | .82 |
| set size × group | 0.55 (1,46) | . 463 | .01 |  | 0.91 (1,46) | .344 | .02 |  |
| cue type × group | 1.48 (2,92) | .233 | .03 |  | 0.35 (2,92) | .705 | .01 |  |
| second delay × group | 1.25 (3,138) | .295 | .03 |  | 0.88 (3,138) | .456 | .02 |  |
| set size × second delay | 2.66 (3,138) | .051 | .06 |  | 0.50 (3,138) | .648 | .01 | .83 |
| set size × cue type | 0.88 (2,92) | .417 | .02 |  | 1.94 (2,92) | .160 | .04 | .79 |
| cue type × second delay | 11.50 (6,276) | <.001 | .20 | .78 | 15.32 (6,276) | <.001 | .25 | .72 |
| set size × second delay × cue type | 0.29 (6,276) | .943 | .01 |  | 1.42 (6,276) | .227 | .03 | .67 |
| cue type × set size × group | 0.56 (2,92) | .576 | .01 |  | 0.15 (2,92) | .863 | <.01 |  |
| cue type × second delay × group | 0.24 (6,276) | .962 | .01 |  | 0.36 (6,276) | .904 | .01 |  |
| set size × second delay × group | 2.61 (3,138) | .054 | .05 |  | 1.63 (3,138) | .184 | .03 |  |
| set size × cue type × second delay × group | 1.62 (6,276) | .142 | .03 |  | 0.90 (6,276) | .494 | .02 |  |

***Note:***df_n_ and df_m_ denote the numerators’ and denominators’ degrees of freedom for the respective *F*-test. ε is the Greenhouse-Geisser estimation of sphericity violations.
